# Actions targeting the integration of peer workforces in mental health organisations: a mixed-methods systematic review

**DOI:** 10.1186/s12888-024-05664-9

**Published:** 2024-03-18

**Authors:** Verity Reeves, Heather McIntyre, Mark Loughhead, Matthew Anthony Halpin, Nicholas Procter

**Affiliations:** The University of South Australia, 5001 Adelaide, GPO Box 2471, South Australia

**Keywords:** Lived experience, Peer support, Mental health, Organisational change management, Organisational learning

## Abstract

**Background:**

Lived experience workforces are one of the fastest growing emerging disciplines in Australian mental health service settings. Individuals with lived and living experience of mental distress employed in mental health services, often referred to as peer or lived experience workers, are widely considered essential for mental health recovery and reform. Despite vast growth of this workforce, concerns remain over the widespread integration of peer workforces to align with recommended movement of healthcare services toward greater recovery-orientated and person-centered practices. Previous research has identified barriers for peer work integration including a lack of clear role definition, inadequate training, and poor supportive organisational culture. Stigma, discrimination and a lack of acceptance by colleagues are also common themes. This systematic review seeks to identify organisational actions to support integration of peer workforces for improved mental health service delivery.

**Method:**

A systematic search was conducted through online databases (*n* = 8) between January 1980 to November 2023. Additional data were sourced from conference proceedings, hand searching grey literature and scanning reference lists. Qualitative data was extracted and synthesised utilising narrative synthesis to identify key themes and findings reported adhere to PRISMA guidelines. The review protocol was registered with Prospero (CRD: 42,021,257,013).

**Results:**

Four key actions were identified: education and training, organisational readiness, Structural adjustments, resourcing and support and, demonstrated commitment to peer integration and recovery practice.

**Conclusions:**

The study identifies actions for mental health service organisations and system leaders to adopt in support of integrating peer and lived experience workforces in service delivery.

## Background

Individuals with lived experience of mental health related distress are a vital component of mental health service reform and recovery [1]. Increasingly, mental health services are employing people who disclose a lived experience of recovery from mental health issues into a variety of roles. Such roles include designated lived experience leaders, peer workers, carer consultants, advocates, educators and policy consultants. This paper focuses on the peer workforce employed in front line mental health service delivery.

Recovery-orientated practice is a generally agreed upon sector approach which emphasises person-centred practices aimed at activating and maximising a person’s self-determination and self-management of mental health and wellbeing [1, 2]. Personal recovery promotes the capacity of a person to live a meaningful life of their choice with or without the presence of symptoms of mental illness [3]. This approach to recovery was developed by and for people with a lived experience of mental health issues to enable them to personally identify and describe their experience beyond the language of clinical diagnosis and treatment [4]. Central to recovery, noting that the journey is different for everyone, is the goal of facilitating choice for activating hope, empowerment, citizenship, advocacy, self-determination and self-management– key principles and values of the lived experience workforce [2].

The growth of peer support practice occurs in conjunction with the growth of consumer leadership. Both are key factors for shaping and re-orientating mental health services and systems [5]. Consumer leadership is evidenced to be beneficial to mental health services however has faced similar barriers in becoming established throughout organisations and systems [6]. It is important to recognise that the sustained development of both the peer workforce at the service level and consumer leadership at governance and policy levels are necessary for systems reform and realising recovery-based care. This has been reflected in the steadily accumulating evidence in support of contributions made by lived experience and peer support work within mental health services [7–9].

Previous reviews of literature have investigated the roles and responsibilities, barriers to and effectiveness of peer support, and the strategies and influences of implementing peer practice across differing global contexts [10–15]. Reviews by both Vandewalle, Debyser [16] and Ibrahim, Thompson [17] established similar influences pertaining to integration barriers identifying organisational culture, role identity and misunderstandings of the role as key themes. Further, Ibrahim, Thompson [17] highlighted the need for initial training and ongoing supervision in designated roles to reduce instances of colleagues’ negative attitudes and support acceptance of peer roles. A review on the roles and responsibilities of peer workers was able to find some consistencies in the role of peer workers, however, was unable to determine consensus across industry literature [10]. A scoping review by Zeng and McNamara [15] investigated strategies utilised to support peer work provision establishing themes of leadership, championing, organisational preparation, training support and development. In further work, Mutschler, Bellamy [11] systematically reviewed barriers and facilitators of peer work implementation using an implementation framework. This review established themes of role clarity, flexible organisational culture and education for both peer and non-peer staff as important elements for implementation [11]. The increased focus on implementing lived experience expertise in mental health services sees a greater need for awareness and support for peer workforces through initial integration and ongoing role support [18, 19].

The provision of appropriate support for the integration of peer services can act as a fundamental element for achieving recovery-orientated practice due to the alignment of their core principles of working side-by-side a person’s needs through recovery [19]. The practice of peer work places high value on human potential and the power of human connectedness leading to greater self-determination and involvement to advance the principles of recovery [20]. Movement toward connectedness and promotion of recovery principles is endorsed through increased employment of peer workers and other lived experience workforce roles in public and non-government services in Australia [21, 22]. Despite ongoing reform efforts to advance a lived experience workforce, recognition and acceptance, misunderstandings and lack of valuing of lived experience expertise remain key concerns [16].

This article focuses on mental health lived experience and peer worker integration to support organisations in operationalising the role effectively and contributing to service development. This review sought to explicate and synthesise organisational actions to support the integration of peer roles in mental health service teams. For the context of this review, the term organisational action refers to acts, steps or mechanisms developed by an organisation to instigate opportunities for learning and effect operational improvement [23, 24]. The term ‘integration’ is utilised to refer to the positive experience of peer workers leading to acceptance by colleagues, ongoing worker retention, and provision of authentic peer support [25]. The term is used in this article as peer workers and non-peer workers regularly interact with each other through the course of performing their duties. That is, a ‘two-way street’ in expecting change across organisations to support and accommodate lived experience workforce development, rather than an adjustment of peer workforces within existing service contexts. This current review differs from previous reviews [11, 15, 17] in its specific focus on tangible actions for organisations to operationalise to support the integration of lived experience practice in service delivery. Emphasis was placed on studies identifying facilitators of mental health peer workforce integration and explicating targeted organisational actions with the research question: “What actions did the organisation take to facilitate peer worker integration into existing mental health service teams?”.

### Aim

The aim of this systematic review was to identify actions or processes organisations have taken which have led to the effective integration of peer roles in mental health service teams.

Researcher perspectives on lived experience workforce related research.

In researching the lived experience workspace, the authors acknowledge that several earlier studies on lived experience roles, performance and outcomes have been led by non-lived experience researchers. This runs the real risk that the research does not reflect lived experience perspectives and recovery principles, and that design considerations and findings are interpreted in clinical terms [26]. Research from this basis can support an implicit co-opting of lived experience roles unless a significant connection to consumer leadership and peer work standards is made in design and interpretation [27]. Lived experience led authorship of research relating to peer workforces is central to this work and is growing in writings of consumer leadership. In acknowledging that this field needs to be increasingly lived experience led, this study focuses on broader organisational actions within literature which can facilitate the successful partnership, integration and sustainability of lived experience by leaders of mental health services, whereby non lived experience leaders can support the growth of the workforce within an allyship framework. This includes a focus on actions that wider workforces can take in creating supportive and understanding environments for/with peer support colleagues [28]. Reflexivity amongst the author team on issues of voice, representation and perspective was assisted by having a mix of lived (ML & MH) and non-lived experience (VR, HM & NP) authors.

## Methods

### Search strategy

The research team developed the search strategy with guidance from an academic librarian. The PICO framework (Population, Intervention, Comparison, Outcome) was utilised to design the search strategy and was conducted in the following databases: Ovid Medline, Ovid Embase, Ovid Emcare, Ovid PsychInfo, Scopus, Web of Science, Ovid Nursing and Joanna Briggs Institute EBP. Variations of Index Headings and keywords were used: mental health, peer support, lived experience, organisational culture, workplace integration. Additionally, hand searching grey literature and consulting reference lists of included articles to seek further potential results. Data search was completed November 2023. Reference managing software (EndNote) was used to manage results and remove duplicates.

### Study selection

**Fig. 1:**
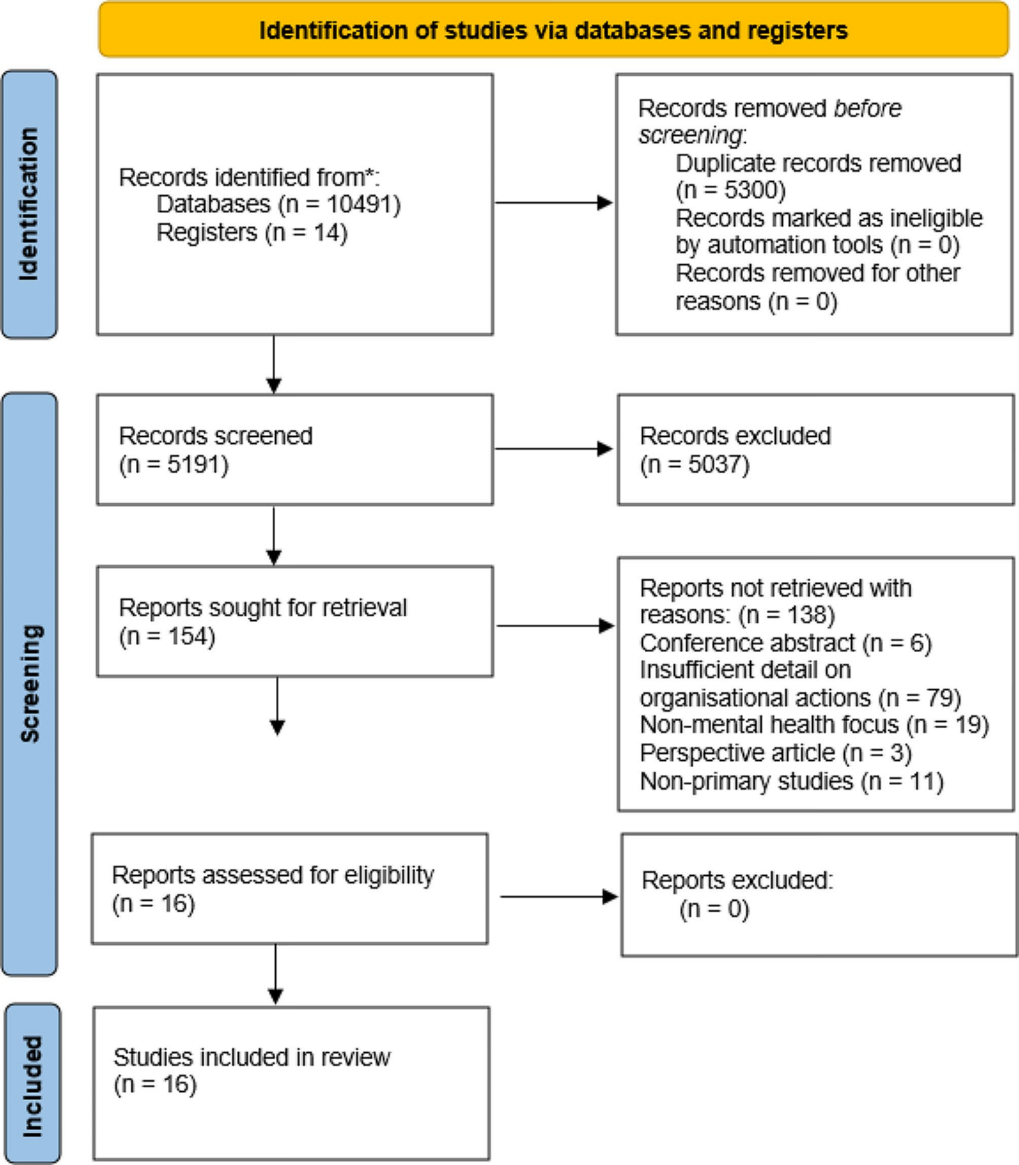
Prisma flow diagram

Initial database searches yielded 10,491 articles. An additional 14 articles were included after scanning reference lists and hand searching grey literature. After exclusion of duplicates (5300), the remaining 5191 articles’ title and abstract were screened against inclusion criteria. In accordance with reporting guidelines for high quality reviews, two researchers (VR and HM) independently conducted title, abstract and full text screening in a blinded process via Covidence [29]. Any conflicts were discussed and resolved prior to moving to the next screening section to achieve 100% agreement for inclusion or exclusion in review. After full text screening, 16 articles remained for inclusion in this review, including qualitative and mixed methods studies. See Fig. 1 for PRISMA flow diagram.

### Eligibility criteria

An eligibility criterion (inclusion/exclusion) was applied during the title, abstract and full text screening to identify relevant articles.

#### Inclusion

Any qualitative or quantitative or mixed method primary study reporting on the integration of peer support or lived experience service delivery roles in mental health services.

#### Exclusion

Any article evaluating the effectiveness of peer support or lived experience roles and services, reviews of literature, proposed studies and grey literature were excluded. Articles about peer integration in other lived experience areas, such as alcohol and other drugs and homelessness were excluded. No language limitations were placed on the search strategy to ensure a comprehensive search was undertaken and all literature relevant to the research question was identified and included in initial screening processes.

### Data extraction and quality appraisal

For each included article, key data extracted included the study aim and design, participant information, summary of organisational actions taken to support integration or sustainability, location and article setting, outcomes of actions taken, if reported, and any factors influencing the integration of peer support workforces into mental health service settings. See Table 1 for study characteristics.

**Table 1:** Study characteristics

Authors and Year	Location	Lived Experience Co-Authorship	Study Design	Sample Size and Characteristics	Data Analysis	Key Findings
Berry, C.; Hayward, M. I.; Chandler, R 2011	United Kingdom	Yes	Qualitative Semi-structured interviews	*N* = 4 2 lived experience workers 2 lived experience managers	Inductive thematic analysis	Organisation to provide support: clear messaging on role of PSW; formal recognition of role through promotion as distinct service; job security; stable working environments; recovery focused teams; Need for ‘readiness’ of PSW employment Highlights need for supervision for role performance, learning and development
Byrne, L.; Roennfeldt, H.; Wang, Y.; O’Shea, P 2019	Australia	Yes	Qualitative	*N* = 29 19 employed in non-peer designated roles, 8 in designated lived experience roles, 2 in lived experience carer roles	Grounded theory	Highlight management and senior management role in creating accepting organisational culture, developing commitment and demonstrating confidence in PSW role. Education for peer supervisors Culturally prepare workplace for introduction of the new role– top-down support.
Byrne, L; Roennfeldt, H; Wolf, J; Linfoot, A; Foglesong, D; Davidson, L; Bellamy, C 2021	United States	Yes	Qualitative	*N* = 132 Interviews [8] and focus groups [14] across five organisations	Grounded theory	Placing peers in positions of senior authority within organisation Whole of workforce training in addition to peer training Peer led supervision Whole of organisation commitment essential to transformation of culture and practice necessary to support effective employment of peers.
Clossey, L.; Gillen, J.; Frankel, H.; Hernandez, J. 2016	United States	Unknown	Qualitative Interviews	*N* = 13 lived experience workers 7 male, 6 female	Grounded theory Inductive approach Action focused research	Organisational culture change through education of the roles– to come directly from the organisation and leadership group Adequately informed supervisors Greater communication of the roles throughout the organisation Championing the role
Franke, C. C. D.; Paton, B. C.; Gassner, L. A. J. 2010	Australia	Unknown	Mixed methods	Survey *n* = 50 Interviews *n* = 25	Thematic analysis	Leadership crucial to how roles initially perceived by staff leading to overall acceptance and positive integration of peer roles Commitment from leaders required in both preparing organisation and supporting peer workers in their roles
Gates, L.; Mandiberg, J.; Akabas, S. 2010	United States	Unknown	Mixed methods– Pilot study Interviews, focus groups and process logs	*N* = 71 23 lived experience workers, 31 non-peer workers, 17 supervisors or directors	Inductive thematic analysis Descriptive statistics Pre/post survey evaluation	Service agency to clearly define peer roles– clearly communicated to all employees and competitive recruitment strategies Consistent policy for all staff Commitment and support from leadership groups
Gillard, S.; Edwards, C.; Gibson, S.; Holley, J.; Owen, K. 2016	United Kingdom	Unknown	Qualitative Comparative case study In-depth interviews	*N* = 89 participants across locations *N* = 10 different case study locations	Descriptive statistics Thematic and framework analysis approach	Equality in pay and valuing peer roles Opportunities for promotion and career development Clear expectations and role clarity Recruiting peers with experience of services they are working within
Hamilton, A. B.; Chinman, M.; Cohen, A. N.; Oberman, R. S.; Young, A. S. 2015	United States	Unknown	Qualitative	Interviews *n* = 19 Focus groups *n* = 8	Thematic analysis guided by diffusion of innovation theory	Detailed pre-implementation planning Sufficient supervision Access to internal and external support Clear expectations for peer roles and on job training for all staff
Kivistö, M.; Martin, M.; Hautala, S; Soronen, K. 2023	Finland	Unknown	Qualitative	Focus Groups total *n* = 22 (*n* = 9 professional staff, *N* = 13 ‘experts by experience’)	Abductive content analysis	Sufficient resourcing and structural adjustment to accommodate lived experience practices and operations. Facilitating culture that values lived experience expertise Facilitating activities to build engagement and buy-in Delivering training and building understanding of peer work. Recruitment of diverse experience and provision of clear role description
Kuek, J.; Chua, H.; Poremski, D. 2023	Singapore	Unknown	Qualitative	Individual interviews *n* = 10 peer support specialists	Thematic analysis	Key actions identified include supportive figures such as leadership ad management roles supportive and inclusive of peer work Clear defined roles to establish boundaries and productive working relationships between peers and non-peers Provision of resources such as offices, materials and funding to conduct peer role.
Mancini, M. A. 2017	United States	Unknown	Qualitative Interviews	*N* = 34 across 10 agencies 23 lived experience workers, 11 non-peers	Thematic analysis	Clear policies, procedures and expectations around introduction of new professional role Clear expectations for outcomes Full inclusion of peer roles into teams Leadership support at executive level and middle management Quality supervision Training for non-peers for the use of peer provided services
Nixdorf, R; Nugent, L; Aslam, R; Barber, S; Charles, A; Mier, L; Grayzman A; Hiltensperger R; Kalha J; Korde, P; Mtei R; Niwemuhwezi, J; Ramesh, M; Ryan, G; Slade, M; Wenzel, L; Mahlke, C. 2022	India Israel Germany Tanzania Uganda	Unknonw	Mixed-methods	Focus groups *n* = 6 (total *n* = 22 participants) Questionnaires *n* = 21	Deductive coding analysis Inductive coding after initial deductive coding to add codes.	Implementing an organisational culture open to learning from each other Prepare staff for changes to teams and service delivery Provision of training to peer workers Organisations establishing and providing clear role definitions and providing adequate resources to undertake the peer role Peers having access to a peer network and ongoing support from organisations to continue to develop
Ramesh, M.; Charles, A.; Grayzman A.; Hiltensperger R.; Kalha, J.; Kulkarni, A.; Mahlke, C.; Moran, G.; Mpango R.; Mueller-Stierlin, A., Nixdorf, R.; Ryan, G.; Shamba, D.; Slade, M. 2023	Germany Israel Tanzania Uganda	Unknown	Qualitative	Focus groups *n* = 12 groups Total of *n* = 86 participants 3 individual interviews	Combination of deductive and inductive thematic analysis	Organisation facilitating activities to promote inclusion of lived experience expertise and reduce stigma associated with mental health Provision of resources to carry out work Clear role definition and organisational expectations Inclusive culture, reasonable adjustments to structures to accommodate peer roles Provision of opportunity to network and create wider peer circles for ongoing external support.
Reeves, V.; Loughhead, M.; Halpin, M.; Procter, N. 2023	Australia	Yes	Qualitative	Individual interviews *n* = 18 All peer workers	Thematic analysis	Prior preparation including education for staff on peer roles and thorough induction of roles to service teams Supervision and opportunity for debriefing, Structural adjustments including clear referral pathways into peer service and regular consultation with peer workforces Sustainable culture changes including leadership support and professional development pathways.
Shepardson, R.; Johnson, E.; Possemato, K.; Arigo, D.; Funderburk, J 2019	United States	Unknown	Qualitative descriptive study Semi structured interviews Exploratory descriptive study	*N* = 24 7 peer support specialists, 6 peer support supervisors, 6 primary care providers and 5 primary care mental health integration workers	Conventional content analysis	Providing peer workers with autonomy to establish roles and responsibilities where needed Supervision from qualified and experienced supervisors Training and upskilling for professional development Commitment from leadership Ensuring peer roles are clear and visible to encourage service use
Stefancic, A.; Bochicchio, L.; Tuda, D.; Harris, Y.; DeSomma, K.; Cabassa, L. 2021	United States	Unknown	Qualitative Semi-structured interviews	*N* = 9 4 lived experience workers, 5 supervisors across three supportive housing agencies	Thematic analysis	Encourage role negotiation and development Multiple supervisors to provide different levels of advice and perspective Encourage professional development Provide opportunity for peers to provide feedback placing values in roles

Quality appraisal was conducted on each included article utilising the mixed methods appraisal tool (MMAT) for peer reviewed journal articles [30]. See Table 2 for evidence of quality appraisal. Risk of bias and quality appraisal was undertaken by two authors (VR and HM) [31]. Duplicated appraisals were compared by both authors to reach consensus.

**Table 2:** Quality appraisal utilising mixed method appraisal tool (MMAT)

Authors and Year	Screening Questions		Methodological Quality Criteria				
	S1	S2	1	2	3	4	5
Berry, C; Hayward M; Chandler R 2011		*	*		*	*	*
Byrne et al. 2019		*	*	*	*	*	*
Byrne et al. 2021	*	*	*	*	*	*	*
Clossey et al. 2016		*	*	*	*	*	*
Franke et al. 2010		*	*		*	*	*
Gates, L; Mandiberg, J; Akabas, S 2010	*	*	*	*		*	*
Gillard et al. 2016	*	*	*	*	*	*	*
Hamilton et al. 2015	*	*	*	*		*	*
Kivistö et al. 2023	*	*	*	*	*	*	*
Kuek et al. 2023	*	*	*	*	*	*	*
Mancini, M. A. 2017	*	*	*	*	*	*	*
Nixdorf et al. 2022	*	*	*		*		*
Ramesh et al. 2023	*	*	*	*	*	*	*
Reeves et al. 2023	*	*	*	*	*	*	*
Shepardson et al. 2019	*	*	*	*	*	*	*
Stefancic et al. 2021	*	*	*		*		*

### Data synthesis

Due to the heterogeneous nature of the included results in this systematic review, a narrative synthesis was selected to identify organisational strategies for lived experience integration and sustainability. Each included article was systematically assessed for its methodological quality, populations, interventions and outcomes. This enabled familiarisation with the data and results of each of the articles included [32]. Authors one and two read articles separately and extracted sections of articles which discussed the process of peer worker integration. Each extracted section was grouped to establish initial overarching descriptive themes. Grouped themes were further reviewed and analysed by the research team to refine and establish subthemes. All authors reviewed the final themes and subthemes identified for any discrepancies and to achieve agreement for final themes reported. See Table 3 for summary of themes associated with reviewed articles.

**Table 3:** Summary of themes and references

Reference	Theme									
	Education and training	Organisational Readiness	Subtheme: Role Clarity	Subtheme: Recruitment and Induction	Structural Adjustment, resourcing and support	Subtheme: Professional supervision	Demonstrated commitment	Leadership	Championing	Commitment and Inclusion
	*(* *n* * = 12)*	*(* *n* ** = 9)**	*(* *n* * = 12)*	*(* *n* * = 7)*	*(* *n* * = 10)*	*(* *n* * = 8)*	*(* *n* * = 15)*	*(* *n* * = 11)*	*(* *n* ** = 5)**	*(* *n* ** = 15)**
Berry, C; Hayward M; Chandler R 2011	*	*	*				*	*	*	*
Byrne et al. 2019	*	*	*		*	*	*	*	*	*
Byrne et al. 2021	*	*	*	*	*	*	*	*	*	*
Clossey et al. 2016					*	*	*	*		*
Franke et al. 2010		*		*			*	*		*
Gates, L; Mandiberg, J; Akabas, S 2010	*		*	*			*	*		*
Gillard et al. 2016			*	*	*		*			*
Hamilton et al. 2015	*	*				*	*			*
Kivistö et al. 2023	*	*	*	*	*	*	*	*		*
Kuek et al. 2023			*		*		*	*	*	
Mancini, M. A. 2017	*		*			*		*		*
Nixdorf et al. 2022	*						*			*
Ramesh et al. 2023	*	*	*		*		*			*
Reeves et al. 2023	*	*	*	*	*	*	*	*	*	*
Shepardson et al. 2019	*	*	*	*	*		*	*		*
Stefancic et al. 2021	*		*		*	*	*			*

## Results

A total of 5191 records were screened, with 154 full-text articles examined resulting in the inclusion of 16 studies. Studies were conducted across 5 main countries including United States (*n* = 7), Australia (*n* = 3), United Kingdom (*n* = 2) with single studies conducted in both Finland and Singapore. Two studies were conducted across multiple countries including India, Israel, Germany, Tanzania and Uganda. Study designs were primarily qualitative (*n* = 13) and mixed-method inquiries (*n* = 3). Studies were appraised for quality and were rated as good quality (*n* = 11), fair quality (*n* = 5) and poor quality (*n* = 0). See Table 2 for quality appraisal.

From the 16 included studies four overarching themes were established including the provision of education and training, organisational readiness, structural adjustment, resourcing and support and demonstrated commitment to peer integration and recovery practice. Within these overarching themes, several sub-themes emerged including role clarity, recruitment and induction, leadership, championing, professional supervision and consistent inclusion which will be discussed in greater detail. See Table 4 for brief description of themes and subthemes.

**Table 4:** Themes and subthemes

Theme	Description and Subtheme
Education and training	The provision of education and training support to non-peer colleagues to improve understanding of the peer role and integrate lived experience support and expertise into consumer recovery journey.
Organisational Readiness	Ensuring organisations are prepared to take on a new role, have a clear understanding of peer roles and practices and where they sit within existing team structures prior to integration into services. This allows distinction from current service delivery roles and opportunity for consultation with existing staff. *Subtheme: Role clarity.* • Alleviates ambiguity for role responsibilities and allows organisations to clearly communicate needs and expectations of peer workers. *Subtheme: Recruitment and induction* • Thorough recruitment and induction processes ensure peer workers possesses appropriate qualities and skillsets to fulfill needs and expectations identified by organisations and integrate into existing service teams.
Structural adjustment, resourcing and support	Provision of resources necessary to undertake the peer role, adjustments within organisational and team structures to include peers and ongoing support for workers. *Subtheme: Professional supervision* • Provision of supervision from a supervisor with lived experience or with a sound understanding of peer workforces contributes to valuing of lived experience expertise in the team.
Demonstrated commitment to peer integration and recovery practices	Demonstrated commitment to peer and recovery values through consistent inclusion, regular consultation and facilitation of activities to improve integration of peer workforces. *Subtheme: Leadership* • Influence of leadership on creating inclusive workplace culture, facilitating opportunities for education, provision of necessary resources for success and promoting value of lived experience expertise. *Subtheme: Championing* • The act of advocating and promoting unique characteristics of peer work to enhance understanding of the role and encourage sustainability through continued use of services. *Subtheme: Consistent inclusion* • Ongoing organisational commitment through consistent inclusion of peer workforces in service delivery, regular use of recovery language, increased visibility and accessibility to peer services.

### Theme 1: education and training

The provision of education and training was found to support integration of peer roles, appearing in 12 of the 16 included articles for this review. Educational approaches are documented in various formats including formal education sessions, the facilitation of open conversations, educational community events, role mapping and increasing employee’s exposure to the work and services peer workers provide. Increasing wider workforces’ understanding of the role was found, in several instances, to reduce employee resistance and discrimination, improve acceptance of the role and facilitate greater engagement with peer support workers and their services [33–36].

The provision of education was found to emphasise the value of lived experience knowledge, provided boundaries for peer workers, and assisted in managing non-peer employee expectations [34, 37]. Byrne, Roennfeldt [38] highlight the provision of education and whole-of-workplace training on lived experience roles is most beneficial when delivered regularly rather than a one off.

Further to the education of employees, research highlights the importance of ongoing training and education for peer workers supporting consistent improvement of service, continuous development of unique skillset and professional growth contributing to sustainability of peer roles [39, 40].

Organisations may support by providing opportunities to gain formal peer support qualifications, create or engage with further learning opportunities, support peers to develop and deliver training for colleagues and provide education on specific workplace policies and procedures to ensure effective integration into teams [41].

### Theme 2: organisational readiness

Ensuring the introduction and integration of peer roles are well planned from the beginning, including clear and distinct role definition, in-depth knowledge of peer roles and practices, sufficient structure to the program contributed to successful integration [38, 42, 43].

Knowledge of previously documented implementation challenges and detailed pre-implementation planning ensures the organisation can adequately prepare and attempt to alleviate potential for negative outcomes [42]. As part of pre-implementation planning, organisations should consider the purpose of establishing and integrating a peer workforce, suggesting if organisations have clarity around their purpose, it will highlight the key areas for organisational development in supporting peer-based practices.

A key component of organisational readiness is consultation with existing staff prior to onboarding peer workers [28, 39, 40, 44]. This approach sees the need for organisations to address concerns of employees and key stakeholders and be conscious and empathetic to their emotions over the change process of introducing new professional staff to a team. This involves open and clear discussions of the role and the provision of education for fellow employees on the nature, role and responsibilities of the peer support position [45], further highlighting the need for organisations to maintain certain flexibility and willingness to make changes in response to feedback provided [46].

#### Subtheme 1: role clarity

Twelve studies highlighted the importance of clear and defined role descriptions provided both to peer support workers and their colleagues for the successful uptake of peer support roles and services. Providing clarity to the peer role was found to alleviate uncertainty and ambiguity for all stakeholders, improved peer confidence in their role and enable a smoother transition to acceptance and understanding of peer worker roles [34, 35, 45]. Crucial for this is acknowledging peer services can vary greatly between organisations, highlighting the importance of establishing and clearly communicating organisational needs and expectations of the role [34]. Further, the provision of a clear role description highlighting the uniqueness of the peer role was found to distinguish peer work from other services and find its appropriate fit within the team and service delivery [44].

#### Subtheme 2: recruitment and induction

The importance of meaningful recruitment and induction ties closely to the organisations need to be prepared to take on the new role. A clear understanding and consideration of the peer role ensures a more robust and thorough recruitment, seeing workers screened for the appropriate qualities and skills required to fill the role and needs of the organisation and service users [38, 47]. Several studies went further than this to highlight the importance of recruiting peer support workers with direct experience of the services they are being recruited to support [39, 48]. Research identified the need for consistent onboarding processes, including structured inductions, highlighting the need to find the best fit for the role as integral to the successful integration into the team [35, 43, 47, 48]. Further, introducing peer concepts for all workforce orientation was found to support integration and overall acceptance of the peer role [38].

### Theme 3: structural adjustments, resourcing and support

In addition to organisation’s establishing readiness for the introduction of peer workers to service teams, ensuring adequate structural adjustments have been made to accommodate the peer role, the provision of resources to effectively undertake the role and ongoing support are crucial. This includes ensuring organisations are flexible and able to make reasonable adjustments for the role, recognising that peer workers shape the role based on their skills and expertise [34, 46]. Further, organisations introducing systems that provide specific information on peer workers including their areas of expertise and instructions on how to ‘book’ specific peers for service users [35].

Commitment to peer practice was demonstrated by providing equal access to opportunities for all staff including fulltime or ongoing work, access to resources (computers, desks, phones, patient records, transport) and staff benefits including annual leave and sick pay [34, 38, 39, 44]. Gates, Mandiberg [47] highlight that applying the same policy for all staff reduced segregation between workers and promoted the inclusion and acceptance of the peer support role. Organisations providing equal opportunity to fair and equal pay places value in the knowledge, expertise and services peer support workers contribute, facilitating inclusion in recovery orientated practice [38].

#### Subtheme 3: professional supervision

Professional supervision was consistently noted across 8 of the 16 articles included in this review. Supervision was found to be effective in supporting integration of peer workforces noting that support from knowledgeable and informed supervisors assisted peers to set and maintain professional boundaries and contributed to feeling valued and accepted in their role [42, 45]. For supervision to be effective, research found that supervisors should have a sound understanding of the peer support role and lived experience workforce [33]. The supervisor should be supportive and champion the value of lived experience knowledge for consumers and the wider recovery movement and encourage role negotiation and development [33, 41]. Lived experience authors raised the context where peer workers often have supervisors who are non-lived experience workers and highlight the importance of ensuring access to peer led supervision for authenticity of practice [38]. Additionally, studies found the need for adequate training of supervisors to ensure they are appropriately supporting and advocating for peer workers [46].

### Theme 4: demonstrated commitment to peer integration and recovery practice

An organisation’s ability to demonstrate commitment to peer integration and recovery practice was noted in various formats by all studies included in this review. Studies noted consistent inclusion of peer workers in teams– where the service is seen as an essential component of recovery and service delivery [38] and facilitates a culture where lived experience expertise is valued by the organisation [35]. Mancini [36] notes need for intentional integration strategies where organisation support and fully endorse the concept and philosophy of peer work and recovery practice.

Further, formal recognition of the peer support role and promotion as a distinct support was seen to legitimise and increase acceptance of the role [37, 41, 44]. Research suggests organisations can demonstrate commitment by supporting peers to access a wider peer network and by hiring multiple peer workers into the service [34, 40].

Teams who acknowledged individual strengths and competencies of peer workers, in addition to ongoing exposure to peer work found greater uptake/use of peer services and increased referrals into the service [42]. Organisations who outwardly displayed positive attitudes and encouragement for the uptake of peer services and invested in prior promotion and advertising of peer services were able to prepare workers and service users for the integration of the service, seeing greater engagement and integration of services [34, 40].

Differing levels of organisational support were reported across each of the articles, seeing the use of strong leadership, robust and inclusive organisational policy, championing of the peer support role and commitment to recovery practice as key approaches to successful integration of peer workforces.

#### Subtheme 4: leadership

Eleven of the sixteen included articles highlighted the importance of strong leadership for the integration of peer workforces. Studies noted the influence leadership has on creating an accepting workplace culture, providing stable working environments and promoting the value and necessity of the peer support role in supporting recovery-orientated practice [34, 43]. Strategies utilised by organisational leaders, as noted previously, include the provision of adequate resources, professional supervision, clear and consistent messaging and role clarity.

Leaders providing clear and consistent messaging through initial integration periods was noted as key to achieving employee commitment and buy-in, an integral component of creating an accepting and inclusive working environment [46]. Leaders consistently demonstrating commitment to peer work practice and articulating clear role and responsibilities was found to increase the likelihood of acceptance by existing employees by reducing stigma or possibility of workers feeling threatened by the new role [36, 46]. This is enhanced by placing peers in senior positions of authority at multiple levels of the organisation including management, executive and board member roles [38, 46]. This provides a clear standpoint for where the organisation sits in regard to the new role, promoting understanding, value and engagement and setting a standard for practice while simultaneously legitimising and justifying the role [46]. It also highlights the organisations commitment to recovery practice [48]. Franke, Paton [49] found that organisational leaders must lead by example to ensure consistent integration. Without support from management, peer support workers found they felt a greater burden of proving their own value to the team.

A key approach to integration included leaders using their platform to highlight the role and potential benefits it can provide to service users, increasing understanding and support from other employees [33, 46]. This was supported by Berry, Hayward [44] noting that the peer support role operates under a strengths-based model and provides leaders an opportunity to promote how unique and distinct the role is and how it differs from other support positions.

#### Subtheme 5: championing

In line with effective leadership as an approach for peer support worker integration is the concept of championing within the organisation. This promotes the unique characteristics of peer support and the distinct value of services whilst facilitating effective integration and acceptance into service delivery teams. This is supported through findings by Gillard, Edwards [37] who highlight that if the peer support role is not emphasized as distinct or unique from other existing support roles, there is little success in its adoption and integration. Championing the role can be implemented via several avenues within the organisation including through support from executive management, promotion of services filtered down through lower-level management or through the appointment of specific peer support champion [38]. The appointment of specific peer support champions are people who have significant understanding of the peer support role, who act as advocates and mentors and who have influence and respect within the workplace [42–44].

Championing through organisational support sees the increase of teamwork opportunities between peer workers and their colleagues, highlighting the unique and positive aspects of peer support. Championing as a strategy to positively influence peer support integration into service delivery stems beyond the education of fellow employees and has proven effective as a strategy to increase understanding and acceptance from mental health service users [33, 38].

#### Subtheme 6: consistent inclusion

Table 2 highlights the fourteen articles identifying the organisations commitment to recovery practice and inclusion of peer support services as a key component for successful integration. This is demonstrated through the consistent use of recovery language in practice, policy and mission statements to assist staff to embody and normalise its use [43]. Inclusion of peer support workers within the workplace increased colleagues exposure to peer support, ensuring increased visibility and accessibility to services, building trust between colleagues, increasing stakeholder buy-in, familiarisation and team cohesion [36, 39, 46]. Inclusion in all staff activities and processes further assisted the integration process, encouraging staff to see peer support as a professional role and a wider part of the team [35, 38]. Recent studies found integration of peer roles was further supported through initiating community and team activities such as working on collaborative projects or community education events to raise awareness of lived experience expertise and value it can bring to service users recovery journey [34]. Further, designated activities were found to shift stigmatizing attitudes toward mental health and assist in facilitating integration of peer services [35].

## Discussion

This article reviewed papers discussing the integration of peer worker roles to identify specific actions taken by organisations to facilitate their integration and improve service delivery. As outlined through the results of this review, actions and approaches centred on education with full support from the organisation being central to the overall integration of peer work services. Four main themes were identified across the literature: education and training, organisational readiness, structural adjustment, resourcing and support and demonstrated commitment to peer integration and recovery practices. Emerging from these themes were sub themes of role clarity, recruitment and induction, leadership, championing, professional supervision and consistent inclusion, each highlighting actions taken by various mental health organisations to effect operational improvement. Despite generating results into themes, there is significant overlap and interconnectedness of themes with each other, noting that education, training and leadership are essential to ensuring organisational readiness, leaders are responsible for allocation of resources and adjusting structures to accommodate and value lived experience expertise. The interdependence of these actions highlights the importance of initiating change throughout all levels of the organisation are needed to improve worker integration.

Studies highlighted the need for initial and ongoing education and training programs for workplaces on the role and value of peer support and how it contributes to systems reform and achieving sustainable cultural change. Crucially, education on peer roles to support cultural change in the workplace, while important, extends beyond valuing the effectiveness of peer support. Cultural change must be combined with education to increase general understanding, reducing instances of stigma and discrimination or ‘othering’ often identified as a barrier to peer support integration [27, 50]. This emphasises the need for strong and consistent communication of the role throughout the organisation and recruitment processes to reduce risk of misunderstanding role responsibilities and duties.

Clear and consistent messaging stemming from the organisation and leadership groups reinforces their commitment to achieving a recovery orientated culture and environment for workers. The findings of this review are consistent with wider literature exploring the impacts of an organisation’s leadership and directives on culture, workplace acceptance and productivity [18, 51, 52]. In particular, the impact leadership has on acceptance of change, how change is implemented and shifts organisational culture in order to facilitate peer worker integration [52].

The results also highlight the need for organisations to take accountability for implementation and sustainability of peer workforces, encouraging systemic change toward great recovery orientated services. Emphasis placed on organisational readiness and support are clear indicators of their centrality in implementing and sustaining peer support. Actions established in this review find the effective integration of peer roles may only be possible in organisations where lived experience expertise is valued and perceived as an essential service that benefits service users. Publicly placing value and importance on peer support as a component of services moving forward influences how peer support roles are received and accepted within working environments. As noted in literature, the shifting of culture within mental health service systems is an essential component and contributor to improving acceptance and sustainability of peer workforces [53].

Consistent with organisations taking accountability and placing value in peer roles is the commitment to providing professional supervision to peer workers. When initially introducing peers into service teams, organisations may not have implemented senior peer roles who may act and provide professional peer supervision to new workers. While it is encouraged to implement peers in leadership positions, while appropriately fulfilling these roles, organisations are encouraged to seek and utilise alternative resources available such as paid peer supervision services offered by state based peak bodies. The Mental Health Coalition of South Australia, for example, provide a paid training program to support professional development for leaders directly supervising peer or lived experience workers [54].

Notably absent in the academic literature is the importance of utilising published lived experience guidelines [53] to assist organisations to develop their workforces in a sustained way over time. Results of this review consistently align with published recommendations and guidelines which highlight the need for organisations to understand lived experience work, commit to workforce development and engage in co-production practices and work together to plan and support mental health system reform. Workforce guidelines, including Australia’s National Mental Health Commission guidelines, in addition to those developed in Queensland and South Australia, encourage organisational change based on guidance from local lived experience leaders and peak bodies [19, 55, 56]. Working from accepted and authentic definitions of lived experience and designated roles avoids role distortion or co-option of peer work values when working alongside clinicians or within mental health services [18]. The widely documented concerns on role clarity have perhaps occurred when services have lacked consultation with guidelines or peer leaders. Individual organisations benefit where there are systems level guidance and infrastructure to facilitate shared understanding, knowhow and collaboration [53]. An integral aspect of workforce development is for organisations to develop a program for change that monitors successful growth and integration. This would include attention on identifying barriers and challenges and evaluating organisational level responses that address them, through continuous improvement [57].

It is also worth acknowledging that considerable literature in this field places heavy focus on barriers or challenges to the integration and uptake of peer workers in mental health services. Through the process of this review, it was recognized that when barriers to peer integration were addressed by organisations, they became facilitators in supporting integration and sustainability of the peer worker role. This includes the provision of infrastructure to support the work of peers, provision of clear role descriptions, setting expectations for workers and supportive leadership.

Results highlight the importance of allyship between workers in assisting peer support practice to thrive and establish itself as an embedded service within mental health service delivery. Allyship, often referred as the actions, behaviours and practices that people in privileged positions take to advocate with and support people who are members of marginalised and oppressed groups [6, 58, 59]. Themes of consistent inclusion, leadership, championing and broader organisational support align with the values of allyship– working in partnership and advocating alongside [60]. This highlights the importance of working alongside lived experience leaders to establish effective mental health systems reform to accommodate lived experience roles within mental health services.

Consistent strong leadership, clear messaging and championing of peer roles plays an integral part in legitimising the services peer practitioners provide, promoting the adoption and adherence to recovery principles, language, attitudes and approach to service delivery. These aspects of leadership need to be augmented by the growth of employed peer leaders and senior practitioners within services, with this being a prominent theme in Australian literature [46] and industry [55, 61]. The promotion of consumer leadership as a key component of recovery orientated practice encourages the shift on a systems level towards a greater more inclusive recovery culture, models of care and service provision [53].

## Conclusions

Inclusion of lived experience expertise within organisations and mental health teams requires the coordination of multiple levels of action and development, with considerable attention given to the construct of service systems to ensure effectiveness and durability in facilitating change for the long term. This places emphasis on system reform and organisational change over accommodation within existing services to ensure effective integration and sustainability of lived experience and recovery orientated services.

### Limitations

This systematic review may be limited by the wide variation of studies, including low confidence in some available evidence due to incomplete reporting, limited study design and inconsistent trial populations. The lack of established RCT’s or quantitative studies published in this area is also a limitation. Further, inconsistent naming of lived experience and peer support search terms across databases may have limited researcher ability to capture all available literature. The broad use of synonyms used for keywords to identify peer work or lived experience can be viewed in the data extraction table.

## Data Availability

Data is provided within the manuscript or supplementary information files.

## Declarations

### Protocol and Registration

This systematic review was conducted in accordance with PRISMA guidelines. The review protocol was registered with Prospero on 1st June 2021 (CRD: 42021257013).
